# Avoiding Pacemaker Implantation by Using Intracardiac Echocardiography During Self-Expanding Valve TAVR

**DOI:** 10.1016/j.jaccas.2025.105061

**Published:** 2025-09-24

**Authors:** Satoki Oka, Syunsuke Matsushita, Akihiro Ikuta, Kazushige Kadota, Shingo Hirao, Tatsuhiko Komiya, Yasushi Fuku

**Affiliations:** a Department of Cardiovascular Medicine, Kurashiki Central Hospital, Kurashiki, Japan; b Department of Cardiovascular Surgery, Kurashiki Central Hospital, Kurashiki, Japan

**Keywords:** aortic stenosis, complete atrioventricular block, intracardiac echocardiography, transcatheter aortic valve replacement

## Abstract

**Background:**

Intracardiac echocardiography during transcatheter aortic valve replacement (TAVR) clarifies the relationship between the bioprosthetic valve and membranous septum (MS), reducing atrioventricular conduction disturbances.

**Case Summary:**

An 82-year-old man with severe aortic stenosis underwent transfemoral TAVR owing to annular calcification; we used a self-expanding 29-mm Evolut FX valve (Medtronic) for the procedure. Computed tomography showed a short MS length, indicating high risk for conduction disturbance. Intracardiac echocardiography revealed that although the valve extended beyond the MS, calcifications at the noncoronary cusp (NCC) and right coronary cusp (RCC) would prevent contact with the muscular septum, avoiding the need for permanent pacemaker implantation (PPI).

**Discussion:**

The conduction pathway and the MS are located between the NCC and RCC. Severe calcification at these sites may reduce the risk of PPI.

**Take-Home Message:**

Significant valve calcification at the NCC and RCC may allow deep implantation during TAVR without PPI.

## Visual Summary

**Figure:**
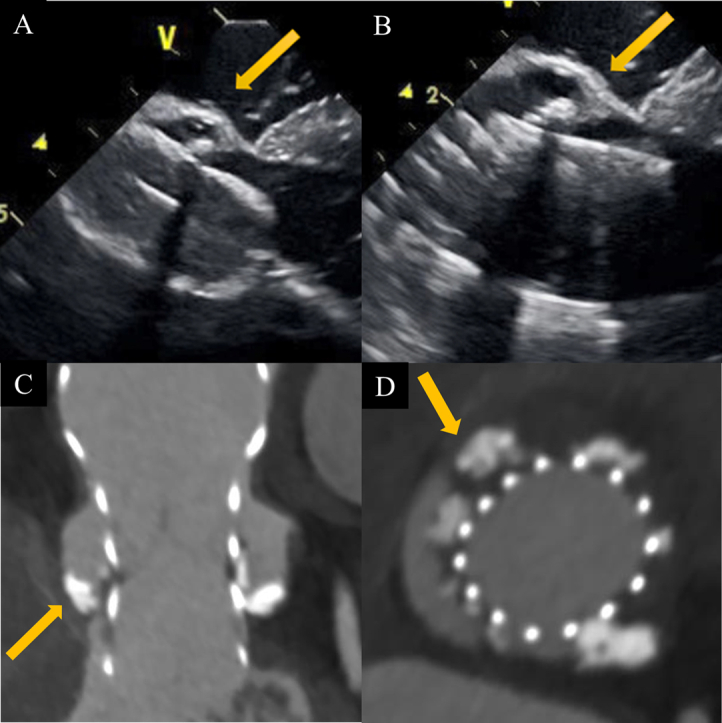

Transcatheter aortic valve replacement (TAVR) has become an established alternative to surgical aortic valve replacement for patients with severe aortic stenosis who are ineligible for or at high risk for surgery.^1^ Because of its promising long-term outcomes, the indications for TAVR have been recently expanded to include patients for whom surgery would pose a lower risk.^2^ However, conduction disturbances requiring permanent pacemaker implantation (PPI) remain a notable complication of TAVR, particularly with the use of self-expanding valves, which are associated with a higher risk of PPI compared with surgical aortic valve replacement.^3^ Anatomically, the conduction system is typically located near the inferior margin of the membranous septum (MS). To reduce the risk of PPI, implantation at a higher location is often necessary, although this may compromise coronary access. Recently, the use of transvenous intracardiac echocardiography (ICE) during TAVR has been reported to reduce the risk of new PPI^4^ because it can clarify the relationship between the self-expanding valve and the MS.

**Take-Home Messages**

• ICE during TAVR helps reduce atrioventricular conduction disturbances.
• Significant valve calcification at the noncoronary cusp and right coronary cusp may allow deep implantation during TAVR without the need for PPI.

At our hospital, ICE is routinely used during TAVR with a self-expanding valve. Here, we report a case in which PPI was avoided despite deep implantation during ICE-guided TAVR.

## Case Presentation

The patient was an 82-year-old man previously diagnosed with aortic valve stenosis. He had recently developed dyspnea assessed as NYHA functional class II and signs of heart failure, leading to hospitalization for further evaluation and treatment.

## Past Medical History

The patient had a history of hypertension and chronic kidney disease (class III).

## Differential Diagnosis

The differential diagnosis included progression of severe aortic stenosis, coronary artery disease, and congestive heart failure.

## Investigations

Electrocardiography showed sinus rhythm with a narrow QRS complex, without bundle branch block. Transthoracic echocardiography revealed a left ventricular ejection fraction of 48% with diffuse mild hypokinesis. Very severe aortic stenosis was confirmed, with an aortic valve area of 0.62 cm^2^, peak velocity of 5.2 m/s, and mean pressure gradient of 70 mm Hg. Mild aortic regurgitation was observed, while no additional significant valvular abnormalities were noted.

**Figure 1:**
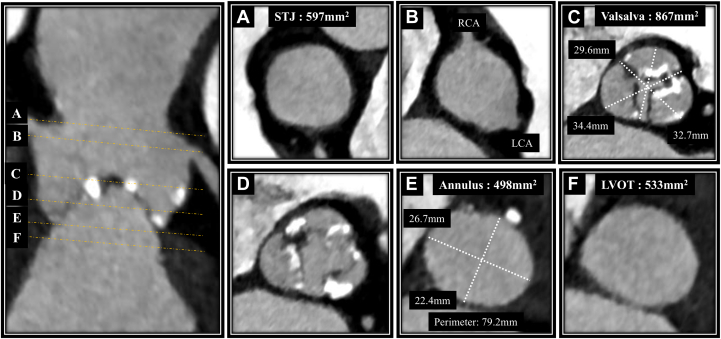
Preoperative Computed Tomography Findings of the Aortic Valve (A) Sinotubular junction (STJ). (B) The ostium of the left coronary artery (LCA) and right coronary artery (RCA). (C and D) Sinus of Valsalva. (E) Annlus. (F) Left ventricular outflow tract (LVOT).

**Figure 2:**
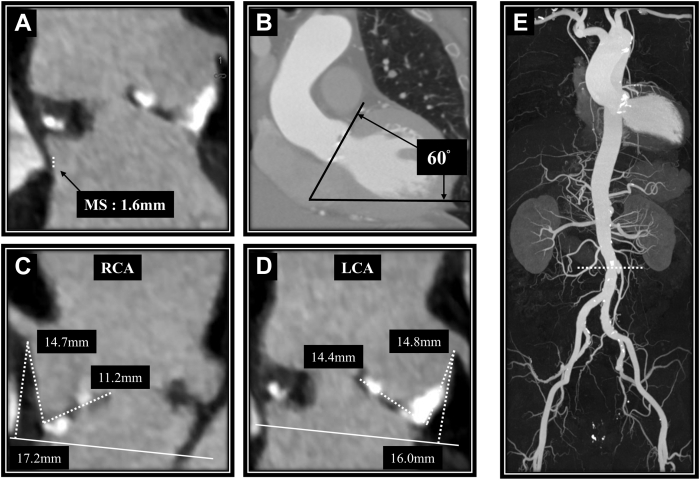
Other Preoperative Computed Tomography Findings (A) Membranous septum (MS). (B) The angle between the aorta and the heart. (C and D) The height of the right coronary artery (RCA) and left coronary artery (LCA). (E) The characteristics of the aorta and lower limb vessels.

Computed tomography scans showed an aortic annulus area of 498 mm^2^ and a perimeter of 79.2 mm, with calcifications on the short-axis side (Figure 1). Severe calcifications were present on all 3 valve leaflets, extending from the right coronary cusp (RCC) into the left ventricular outflow tract. The MS measured 1.6 mm, indicating a high risk for new PPI. The aorta and lower limb vasculature exhibited minor plaques, but their characteristics and diameters were deemed suitable for a transfemoral approach (Figure 2). After discussion with our heart team, we decided to perform transfemoral TAVR using a self-expanding 29-mm Evolut FX valve (Medtronic) under ICE guidance.

## Treatment

**Figure 3:**
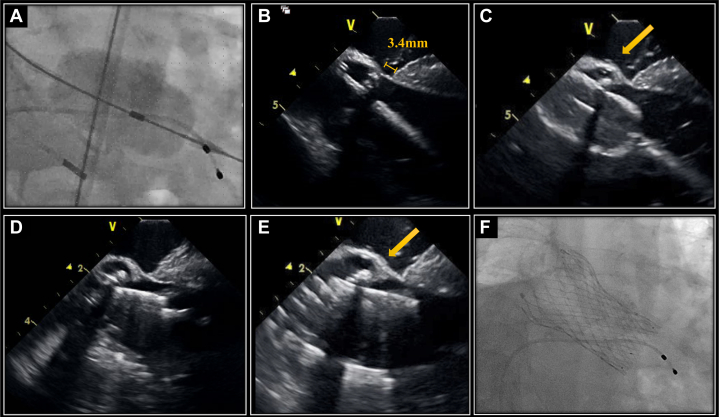
Fluoroscopic and Intracardiac Echocardiography Images During Procedures (A) Fluoroscopic view of a predilation using a 22-mm Inoue balloon. (B) Intracardiac echocardiography (ICE) image of membranous septum (MS). (C) ICE image showing calcification between the balloon and the MS during predilation. (D) ICE image at the point of no recapture. (E) ICE image showing calcification between Evolut valve and the MS after valve deployment. (F) Fluoroscopic view of a valve in the cusp overlap view.

During predilatation with a 22-mm Inoue balloon, ICE revealed calcifications between the balloon and the MS. The Evolut valve was subsequently deployed. ICE indicated that the lower edge of this valve extended significantly beyond the MS, but the calcifications observed during balloon aortic valvuloplasty prevented direct contact between the Evolut and the muscular septum (Figure 3). Paravalvular leakage was mild. Despite the deep implantation relative to the short length of the MS, atrioventricular conduction was preserved, and the procedure was successfully completed on the first deployment.

## Outcomes and Follow-Up

**Figure 4:**
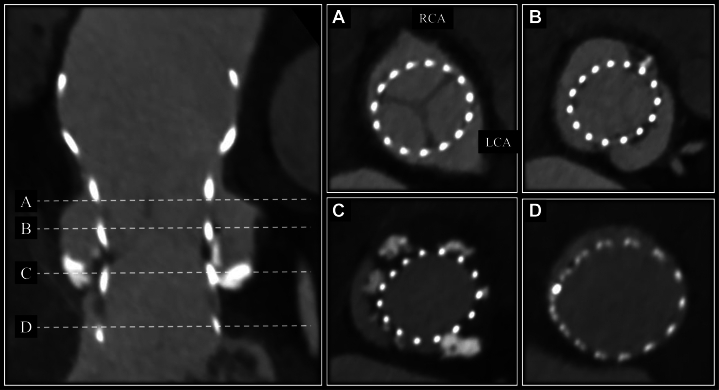
Postoperative Cardiac Computed Tomography Findings (A) Near the sinotubular junction. (B) Upper level of the sinus of Valsalva. (C) Lower level of the sinus of Valsalva. (D) Left ventricular outflow tract.

**Figure 5:**
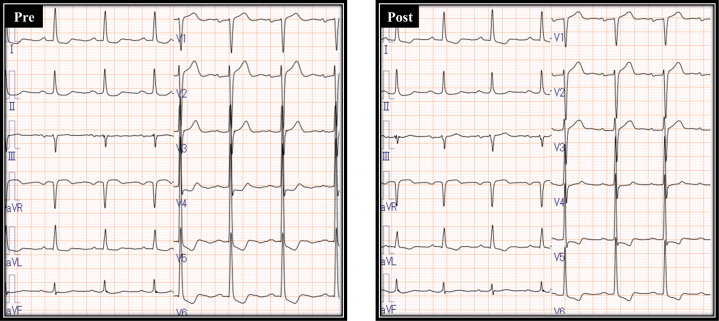
Comparison of Electrocardiogram Findings Before and After Transcatheter Aortic Valve Replacement

Postoperative computed tomography confirmed significant calcifications extending from the noncoronary cusp (NCC) to the RCC (Figure 4). Electrocardiography showed no significant changes between the preoperative and postoperative periods (Figure 5). Echocardiography before discharge showed trivial to mild paravalvular leakage and a mean pressure gradient of 12 mm Hg. The patient was discharged without requiring PPI. Even after 1 year of follow-up, the patient has still not needed PPI.

## Discussion

**Figure 6:**
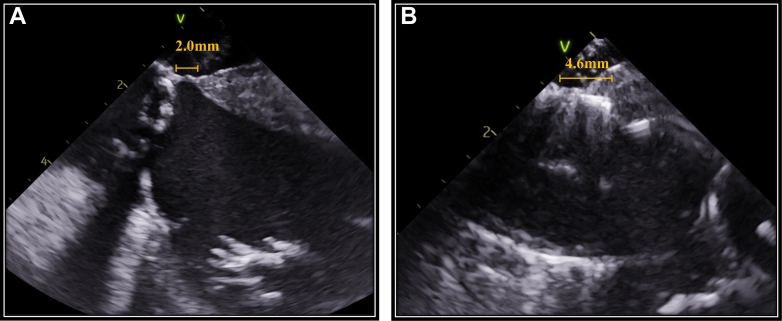
Intracardiac Echocardiography Findings in a Comparison Case With Valve Contact to the Muscular Septum (A) Preprocedural intracardiac echocardiography (ICE) image showing measurement of the membranous septum length. (B) ICE image after valve deployment showing that the prosthetic valve extended beyond the membranous septum and directly contacted the muscular septum.

Risk factors for a new PPI after TAVR include right bundle branch block, short MS length, and implantation depth.^5^ ICE is particularly specialized in real-time navigation of the MS when compared with transesophageal echocardiography. It enables accurate evaluation of the positional relationship among the MS, muscular septum, and implantation depth during the procedure, ensuring correct placement within the MS. This allows operators to intentionally deploy the valve deeply, up to the edge of the muscular septum, without contacting it. Additionally, ICE guidance helps operators decide whether recapture is necessary in cases of deep implantation, based not only on markers of implantation depth but on real-time spatial visualization of the valve-septum relationship. In fact, when the prosthetic valve extends beyond the MS and directly contacts the muscular septum, complete atrioventricular block can occur. In another patient, ICE revealed such direct contact between the valve frame and the muscular septum, resulting in complete atrioventricular block and the need for PPI (Figure 6). This comparison in outcomes highlights the clinical importance of avoiding muscular septum contact and underscores the value of ICE guidance in minimizing conduction disturbances during deep valve implantation. This approach has been reported to result in a lower pacemaker implantation rate, even in patients with a pre-existing right bundle branch block.^6^

The difference between MS length and implantation depth has been identified as a predictor of PPI risk.^7^ However, deep implantation beyond the MS does not always necessitate pacemaker implantation. In our patient, severe calcifications in the RCC and NCC displaced the prosthetic valve away from the muscular septum, thereby sparing atrioventricular conduction. Additionally, it has been reported that a larger left ventricular outflow tract relative to the annulus size is associated with a reduced risk of atrioventricular block.^8^ Thus, ICE-guided TAVR may allow deep implantation of the prosthetic valve beyond the MS without conduction disturbances in patients with extensive calcifications in the RCC and NCC and those with a large left ventricular outflow tract. In our patient, without ICE, the gold marker of the Evolut FX would have indicated an implantation depth of approximately 4 mm, which, in the context of an MS length of only 1.6 mm on preprocedural computed tomography, might have prompted a recapture and repositioning. ICE guidance can help avoid unnecessary procedural steps and contribute to a safer and more efficient TAVR procedure.

As TAVR expands to younger, lower risk patients, deeper valve implantation is often required to achieve optimal coronary access and facilitate future valve-in-valve procedures. However, this approach carries an increased risk of conduction disturbances requiring PPI. Our findings indicate that ICE-guided TAVR may enable safer and more accurate deep implantation by providing precise visualization of the anatomical relationship between the prosthetic valve and the MS in selected cases.

## Conclusions

We report a case of deep implantation of the Evolut FX during ICE-guided TAVR, successfully avoiding PPI despite the Evolut valve extending beyond the MS; severe calcifications in the native valve prevented conduction disturbances.

**Visual Summary:**
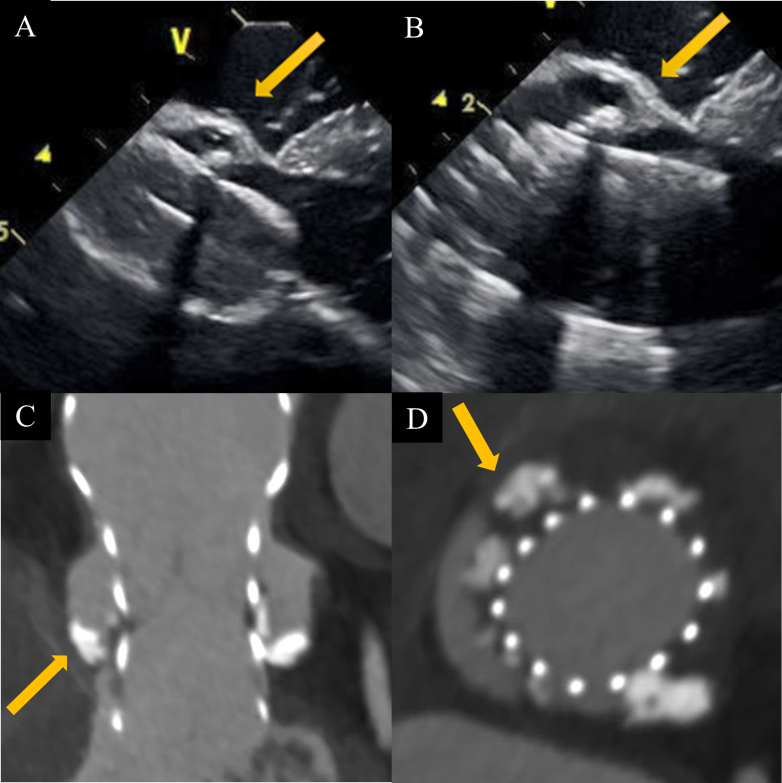
Calcification Between Bioprosthetic Valve and Membranous Septum Demonstrated by Intracardiac Echocardiography and Computed Tomography (A) Intracardiac echocardiography (ICE) image showing the calcification between the balloon and the membranous septum (MS) during predilation. (B) ICE image showing the calcification between the Evolut valve and the MS after valve deployment. (C and D) Computed tomography showing the calcification between the Evolut valve and the MS.

## Funding Support and Author Disclosures

Dr Fuku is a proctor for transfemoral TAVR for Medtronic. All other authors have reported that they have no relationships relevant to the content of this paper to disclose.
